# Identified needs of the very old accessing mental health services in Australia and New Zealand

**DOI:** 10.1177/10398562251407049

**Published:** 2025-12-24

**Authors:** Roderick McKay, Gary Cheung, Anne Wand

**Affiliations:** School of Medicine, The University of Notre Dame Australia, Darlinghurst, NSW, Australia; School of Psychiatry, University of New South Wales, Sydney, NSW, Australia; Department of Psychological Medicine, School of Medicine, Faculty of Medical and Health Sciences, The University of Auckland, Auckland, New Zealand; School of Clinical Medicine, Discipline of Psychiatry and Mental Health, Faculty of Medicine and Health, University of New South Wales (UNSW), Sydney, NSW, Australia; Specialty of Psychiatry, Faculty of Medicine and Health, The University of Sydney, Sydney, NSW, Australia

**Keywords:** very old age, old age psychiatry, HoNOS, routine outcome measurement, needs assessment

## Abstract

**Objectives:**

To examine identified needs of ‘very old’ people accessing Australian and New Zealand specialist mental health services and consider service delivery implications.

**Method:**

Examination of Australian and New Zealand routine outcome measure data from admissions to specialist mental health services. Comparison of data for over 16,500 admissions of people aged 85 years or over with people aged 65–84 years.

**Results:**

Those aged over 85 have higher total HoNOS65+ scores, driven by raised impairment and social problem subscales, without reduction in symptom subscales. Increased problems were identified in aggression/agitation, cognitive impairment, physical health and activities of daily living items but reduced prevalence of substance related problems. Whilst magnitudes vary, trends are consistent across countries, and across ambulatory and inpatient settings. On admission to Australian inpatient settings, very old age is associated with >30% of people requiring nursing assistance (measured by RUG-ADL), although <10% required two-person assistance on any domain.

**Conclusions:**

Very old age at admission to mental healthcare is associated with increasing complexity and nursing support needs, without reduced psychiatric symptoms. This requires consideration in models of care and staff capabilities. The very old with greatest aged-related support needs may be excluded from specialist inpatient care.

## Introduction

Very old age is associated with increasing rates of mental health conditions after ‘peak mental health’ in earlier old age,^1^ but little is known about mental healthcare for the very old. Health status, environmental and societal contexts change across older age,^1,2^ but characteristics of the ‘very old’ who receive mental healthcare are poorly understood, beyond changes in diagnostic prevalence; with reported increasing rates of neurocognitive and mood disorders, and reducing primary psychotic diagnoses.^3^ What is meant by ‘very old’ is debated, as is the use of age as a marker, but thresholds between 75 and 85 years are common in studies of the ‘very old’,^4^ with a threshold of 85 years used in this paper.

Australian and New Zealand mental health services for older people have many similarities. With shared relevant medical colleges, services in both countries are relatively small, public, secondary level multidisciplinary services, with defined geographical catchments.^5,6^ Most services in Australia are funded through mental health.^7^ New Zealand services have significant funding variation from both mental health and geriatric medical sources,^5,8^ greater involvement with people with cognitive impairment,^5,6^ but no national service for people with behavioural or psychological symptoms of dementia.^6^

Several factors suggest very old people may not be considered a ‘core’ focus of mental health services in Australia or New Zealand. Very old people have no presence in national mental health strategies^9^ and have never been the focus of national action, even for suicide prevention^10^ despite very high rates suicide in the very old.^3^ The most recent national study of Mental Health and Wellbeing in Australia excluded those aged 85+.^11^ Specialist mental healthcare access for very old people in Australia has declined over time despite increased mental health related emergency department presentations and hospital admissions without specialist mental health input.^7^ Inpatient funding per day is lower for older people.^12^ Together, these data suggest assumptions that the very old are less severely unwell or in need of specialist mental healthcare.

With increasing numbers of very old people,^1,3^ it is imperative we better understand the very old who access specialist mental healthcare, and how they may vary from the ‘younger old’. These data are required to inform care of very old adults accessing specialist services and support advocacy and planning.

Both Australia and New Zealand routinely collect the Health of the Nation Outcome Scales for Elderly People (HoNOS65+)^13^ at admission of older people to community and inpatient mental healthcare. Australia also collects the Resource Utilisation Groups-Activities of Daily Living Subscales (RUG-ADL)^14^ on entry to inpatient older peoples mental healthcare. The aim of this study was to determine whether there are any differences in the complexity and severity of mental health problems, measured by HoNOS65+, in the very old (85 and older), compared with younger old adults (65–74 and 75–84) accessing specialist mental healthcare in Australia and New Zealand. In addition, changes with age in RUG-ADL data on dependency were examined for Australia.

## Method

This is a retrospective observational study of routinely collected mental health data in Australia and New Zealand from 2019 to 2023; ‘Cleansed’ summary HoNOS65+ and RUG-ADL data are publicly available from the Australian National Outcomes and Casemix Collection (NOCC).^15^ New Zealand HoNOS65+ data are available from the New Zealand Programme for the Integration of Mental Health Data.^16^ Data collection occurs at every new admission and will include multiple collections from some individuals.

### HoNOS65+

The HoNOS65+ is a clinician-rated measure with twelve 5-point items (scored 0 to 4, with higher scores indicating more severe problems), with guidance that scores of ⩾2 on any item are ‘clinically significant’.^17^ Both countries report HoNOS65+data as a total score, individual items and subscales (behaviour, impairment, symptoms, social).^16,18^ Total score captures overall complexity of person (and closest correlation with funding) and subscales inform which areas of function contribute most to this complexity, while analysis of the proportion of people with significant scores on individual items provides a better understanding of the breadth of problems to be considered.

The following summary HoNOS65+ data were extracted for admissions to community and inpatient mental healthcare across the three age groups (65–74, 75–84, 85+) in both countries:

(i) Mean total HoNOS65+ score, as a measure of overall complexity or severity of problems identified;
(ii) Mean HoNOS65+ subscale scores, as measures of overall complexity or severity of domains of problems; and
(iii) Proportion of the HoNOS65+ items with a score ≥2, as a measure of the prevalence of problems identified.

The very small percentage^15,19^ of admissions of older people rated using the HoNOS (a closely related but different measure) were excluded.

### RUG-ADL data

The RUG-ADL is a clinician-rated measure of what a person does and assistance required across four activities of daily living (ADL): eating, transfers, toileting and mobility.^14^ Each activity is rated on a subscale with a rating for ‘requires hands on assistance’ [of one person] and ‘two persons assist’. It provides additional information to the HoNOS65+ regarding functional loss related needs.

Summary RUG-ADL data were extracted for admissions to Australian mental health inpatient care. The glossary was used to identify scores indicative of requiring either one or two-person assistance with ADL care.

## Results

### Mean total HoNOS65+ and the 4 subscale scores

Across both Australia and New Zealand, the mean total HoNOS65+ score increased with age in inpatient and community admissions (Table 1). Across all settings, the increase was largely attributable to the increase within the impairment subscale, with smaller increases in social problem subscale. In New Zealand community settings, symptom subscale scores also increased with age.

**Table 1.** Mean total HoNOS65+ and the 4 subscales scores.

	Age	Inpatient					
Country	Years	*N*	Total score mean	Behaviour subscale^a^ mean	Impairment subscale^b^ mean	Symptoms subscale^c^ mean	Social subscale^d^ mean
Australia	85+	1966	16.9	2.7	3.9	5.5	5
	75–84	6634	15.7	2.7	3.3	5.5	4.4
	65–74	11,282	15.1	2.8	2.7	5.5	4.3
	All 65+	19,882	15.5	2.8	3	5.5	4.4
New Zealand	85+	398	16.5	2.4	4.5	4.4	5.3
	75–84	1303	16.1	2.4	3.9	4.7	5.1
	65–74	2495	14.7	2.4	3.0	4.8	4.6
	All 65+	4196	15.3	2.4	3.4	4.7	4.8
Community							
Australia	85+	10,548	12.9	1.5	3.9	3.6	4
	75–84	22,733	11.9	1.4	3.3	3.6	3.7
	65–74	29,198	11.2	1.5	2.6	3.6	3.6
	All 65+	62,479	11.8	1.4	3.1	3.6	3.7
New Zealand	85+	3597	12.7	1.4	4.3	4.3	4.2
	75–84	6621	11.8	1.3	3.6	3.6	3.7
	65–74	6895	10.8	1.4	2.7	2.7	3.3
	All 65+	17,113	11.6	1.3	3.4	3.4	3.6

^a^Includes items of aggression, self-harm, substance abuse.

^b^Includes items of cognitive impairment and physical disability.

^c^Includes items for hallucinations and delusions, depression, other symptoms.

^d^Includes items for personal relationships, overall functioning, residential problems and occupational problems.

Within each age group, impairment subscale scores were similar across inpatient and community settings in Australia and New Zealand. All other subscale scores were higher in inpatient settings than community settings across both countries.

### Proportion of the 12 HoNOS65+ items with a score ≥2

The prevalence of clinically significant problems related to aggressive/agitated behaviours, cognition, physical illness and ADLs increased with age across all settings; with a reduction in identified problems related to substance abuse (Tables 2 and 3).

**Table 2.** Proportion of the 12 HoNOS65+ items with a score ≥2 at inpatient admission.

Country	Australia			New Zealand		
Age	65–74	75–84	85+	65–74	75–84	85+
Item 1	41%	45%	49%	38%	47%	50%
Behaviours						
Item 2	27%	26%	27%	19%	17%	15%
Self-harm						
Item 3	17%	10%	7%	13%	6%	4%
Substance abuse						
Item 4	37%	49%	60%	45%	66%	70%
Cognitive						
Item 5	47%	56%	66%	49%	58%	72%
Physical						
Item 6	45%	43%	44%	44%	44%	41%
Hallucinations/delusions						
Item 7	63%	63%	63%	45%	44%	39%
Depression						
Item 8	67%	67%	67%	62%	60%	55%
Other problems						
Item 9	42%	38%	36%	46%	47%	43%
Relationships						
Item 10	41%	49%	60%	51%	62%	72%
ADLs						
Item 11	23%	24%	28%	23%	28%	27%
Living conditions						
Item 12	29%	29%	34%	26%	32%	31%
Occupations

**Table 3.** Proportion of the 12 HoNOS65+ items with a score≥2 at community admission.

Country	Australia			New Zealand		
Age	65–74	75–84	85+	65–74	75–84	85+
Item 1	21%	25%	33%	20%	27%	35%
Behaviours						
Item 2	10%	9%	8%	8%	5%	4%
Self-harm						
Item 3	10%	5%	2%	8%	3%	2%
Substance abuse						
Item 4	32%	47%	59%	37%	59%	73%
Cognitive						
Item 5	52%	61%	69%	48%	57%	69%
Physical						
Item 6	20%	22%	25%	19%	22%	24%
Hallucinations/delusions						
Item 7	49%	49%	48%	42%	35%	27%
Depression						
Item 8	55%	54%	52%	56%	52%	46%
Other problems						
Item 9	37%	34%	31%	33%	31%	30%
Relationships						
Item 10	35%	43%	53%	37%	54%	68%
ADLs						
Item 11	15%	13%	14%	11%	13%	15%
Living conditions						
Item 12	26%	28%	31%	19%	22%	25%
Occupations						

Across ages, the prevalence of problems related to suicidal behaviours and depressed mood in Australian inpatient and community settings was higher than New Zealand. Australian inpatient and community settings had lower rates of problems related to cognitive impairment than New Zealand cohorts.

The prevalence of clinically significant problems related to depressed mood was relatively similar across the three age groups in Australian inpatient and community settings but reduced with increasing age in New Zealand inpatient and community settings.

### Australian RUG-ADL: need for assistance with ADLs

Table 4 shows the rates of 1-person and 2-persons assistance for bed mobility, toileting, transfer and eating increased with age in Australian inpatient settings, suggesting domains of functional impairment increased with age.

**Table 4.** Assistance required identified in RUG-ADL data at Australian inpatient admission.

Age	*N*	Item 1 – Bed mobility	Item 2 – Toileting	Item 3 – Transfer	Item 4 – Eating
One-person assistance^a^					
85+	2053	24%	38%	30%	18%
75–84	6480	15%	25%	19%	12%
65–74	10,535	8%	14%	10%	7%
All 65+	19,068	12%	20%	15%	2%
Two-person assistance^b^					
85+	1940	4%	7%	6%	4%
75–84	6424	3%	5%	4%	2%
65–74	10,636	1%	3%	2%	2%
All 65+	19,000	2%	4%	3%	2%

^a^For bed mobility, toileting and transfer, score = 3, For eating, score = 2.

^b^For bed mobility, toileting and transfer, score = 5, For eating, score = 3.

## Discussion

The results of this analysis demonstrate that the very old have greater complexity of identified problems at admission to mental healthcare due to increasing problems in social and impairment domains, without reduction in severity of ‘mental health symptoms’. In Australian inpatient settings, this is accompanied by increasing dependency on nursing care for ADLs, although infrequently requiring two-person assistance. Within this ‘big picture’, there are differences in which problems contribute to complexity. There are more identified problems related to aggression/agitation, cognitive impairment, physical health and ADLs, but reduced rates of substance related problems. This raises questions regarding current access to mental healthcare, models of care and required staff capabilities in all parts of mental health services working with older people.

### Access

These findings could be used to counter misconceptions impeding older peoples’ access to mental healthcare and to advocate for appropriate related guidance. Rates of access to specialist mental healthcare in New Zealand increase from age 65,^20^ whilst remaining significantly below access of younger people. In Australia, access declines with increasing older age.^7^ Factors that impede timely access to appropriate mental healthcare for this population are not well defined. Hypotheses include stigma related to age and mental illness within health and mental healthcare^3^ impacting referrals and complexity affecting decisions regarding care settings or providers.^7^ Low numbers of very old people requiring two-person assistance suggests high nursing dependency may also be a barrier to specialist mental health admission; together with varying interpretations of the role of public mental health services for people with dementia or in residential aged care.^5,6,21^ It is also possible that frequent use of terms like ‘most severe’, ‘urgent’ or ‘acute’ in public mental health service role descriptors,^22^ triage and referral systems^23^ potentially discourage referral of older people who present differently to younger adults,^3^ combined with the influence of ‘intuition’^24^ on referral processes.

Differences between Australia and New Zealand also require consideration regarding their implications for access. Australian settings had higher prevalence of problems related to suicidal behaviours and depression; and lower rates of problems related to cognitive impairment. Presumably, considering the higher prevalence of cognitive problems at admission to New Zealand mental health services, it is likely that more very old people with cognitive impairment and mental health problems in Australia are cared for by other services such as general practice, geriatric medicine, or dementia specific services; with varied concurrent access to specialist mental healthcare.^25^ There were also differences in trends with age, with the problems related to depressed mood increasing with age in Australian settings but reducing in New Zealand. This suggests that if the availability and pathways to clinical resources are unchanged, decisions to admit people with one group of problems may limit access to others. Findings of this study, combined with identified changes in access over time in mental healthcare in Australia,^7^ remind us that such decisions have real world outcomes including increased need for emergency department and acute general hospital care. This seems a compelling reason for planners to act on the major gap between projections of the needs of older people^22^ and current resource availability.^26^

### Staff capabilities

Targeted resources for the very old require an appropriate workforce.^27^ These findings suggest that staff working with the very old who are being assessed for or receiving mental healthcare, require specialist capabilities. That is not just skills, but the ability to apply these without ageism, and match them to individual needs. Given that there is a worldwide shortage of clinicians of all disciplines^3^ trained specifically in providing mental healthcare for older people, it appears likely that workforce solutions are needed beyond training more specialised staff. There is a need to identify sets of skills (and attitudes) clinicians may require for particular roles they have working with the very old,^3^ such as assessment in emergency departments, crisis teams, or geriatric teams. It will also require consideration of how to best use emerging workforces such as lived experience workers or clinical assistants to meet the needs of the very old person. Adaptations are also required in approach and therapies.^3^ Novel ways of bringing together the expertise of various clinicians from traditionally different teams may be needed to meet an individual’s needs: such as specialist older peoples mental health clinicians ‘buddying’ with adult mental health or geriatric clinicians for particular purposes. This highlights the importance of exploring what models of care will work best for the very old with mental ill-health; a challenge given limited data on the effectiveness of various psychiatric services for older people in general.^3^

### Models of care

These data suggest significant work is required regarding optimum models for working with the very old with mental healthcare. It is clear that ‘mental health’ problem severity does not reduce in very old age, so specialist mental healthcare is needed. However, it is also clear that these care needs are increasingly complicated by co-morbid problems commonly managed by geriatric medical services. These data demonstrate that collaboration between these services is increasingly important in very old age.^3^ However, it also raises questions about how and where this care should occur and whose ‘responsibility’ it may be. Healthcare systems may need to change to allow any (or an identified range of) teams to be responsible for ensuring the holistic care of an older person with mental health problems.

In community contexts, a collaborative model would require support from intake systems to allow ‘no wrong door’ referrals; and innovative models to bring skill sets to individuals in an integrated, timely manner; whilst keeping the person, carer and general practitioner at the centre of care.^3^

In inpatient contexts, the limited number of older people requiring two-person assistance accessing specialist mental health inpatient care in Australia raises questions regarding access to care for those who are most physically dependant and suggests dependency may be one of the contributors to the high rate of mental health admissions of the very old to general hospital beds.^7^ How do the shorter lengths of stay and more physical health focused practices of such settings fit with the needs of older people with acute mental illness? When may this approach be most appropriate, or not? Are better partnerships required with community and residential aged care providers to facilitate rapid outreach responses which enable community mental healthcare to reduce admissions? And how do these findings align with older persons’ mental health inpatient services which currently have lower bed day funding than ‘adult’ units?^28^

The degree of flexibility required ‘across siloes’ poses challenges for ‘across age’ mental health services unless there is strong leadership, and investment, to maintain sub-teams with specialist capabilities; active partnerships with aged-specific partners; and intake systems able to recognise, cope with and address the differing presentations of the very old requiring specialist mental healthcare.

This study demonstrates the value of using routinely collected measures in varying ways to improve understanding of the needs of people seeking mental healthcare. Diagnoses are important (and are also known to change in pattern with increasing age^3,7^) but these data enable consideration of the problems and needs of the person to be addressed alongside treating the presenting mental illness.

## Limitations

The limitations of this study relate to the nature of tools used to identify problems, and data organisation in public reporting. Examining cross-sectional mandated data collections provided data from a large number of admissions of very old people, from collections with estimates of compliance over 90%^6^; but meant using summary data that has been pre-processed.^15,19^ It is therefore not possible to account for the effects of missing data, to undertake inferential statistics, or draw firm causal conclusions. Due to bi-national differences in data availability, and study scope, neither diagnostic nor demographic data beyond age were extracted at the time of this study to enable cross-national examination of these factors against HoNOS65+.

Psychometric factors are unlikely to explain trends, with no known changes in scale properties with age^29^; and alignment of findings of increasing impairment with age (as measured on the HoNOS65+) and increasing dependency (as measured on the RUG-ADL). The ‘best’ way of constructing HoNOS65+ subscales has been debated,^29^ with this paper using the most widely reported structure, also used in binational reporting.

Differences in clinician identification of problems with increasing age cannot be excluded; given known impacts of ageism and training limitations on mental health clinicians.^30^

## Future research directions

The limitations of this study highlight the importance of future research to better understand:

• The *causes* of the differences in needs of very old people compared with younger people at admission to mental healthcare, including impacts of healthcare provider stigma on decisions regarding eligibility for care.
• Factors beyond age affecting the needs of individuals entering mental healthcare, such as generational differences in stigma or healthcare utilisation, gender, mental illness or comorbid conditions
• The impact of dependency on access to inpatient mental healthcare, including through exploring associations between HoNOS65+ and RUG-ADL data
• Outcomes of care in the very old.

## Conclusions

There has been inadequate attention paid to the needs of the very old accessing mental healthcare. This study clearly identifies they have different needs to even the ‘young old’, let alone the general population. Because of the population structure, with relatively few ‘very old’; it is too easy for these needs to be hidden when data are reported for all older people; and services planned based on this data.

Awareness that there are differences in need is one part of challenging clinicians’ perception of what is ‘normal’ in very old people accessing mental healthcare. This may have an impact on the cognitive biases known to effect clinician assessments and decision-making regarding older people.^24^ Resilience to the effects of chronic illness probably increases with age,^1,3^ but the prevalence of multiple chronic illnesses increases more.^1^ The very old need services and clinicians able to recognise both the strengths and vulnerabilities of older people; equipped with the skills and service models to meet their needs. New ways of working are required; and given our ageing populations, the need is pressing.
